# Effects of antibody to receptor activator of nuclear factor κ-B ligand on inflammation and cartilage degradation in collagen antibody-induced arthritis in mice

**DOI:** 10.1186/s12952-014-0018-0

**Published:** 2014-12-12

**Authors:** Sakie Funato, Akihiro Matsunaga, Koei Oh, Yoichi Miyamoto, Kentaro Yoshimura, Junichi Tanaka, Dai Suzuki, Risa Uyama, Hiroaki Suzuki, Kenji Mishima, Masanori Nakamura, Osamu Namiki, Kazuyoshi Baba, Katsunori Inagaki, Ryutaro Kamijo

**Affiliations:** Department of Biochemistry, Showa University School of Dentistry, 1-5-8 Hatanodai, Shinagawa, Tokyo, 142-8555 Japan; Department of Orthopaedic Surgery, Showa University School of Medicine, Tokyo, Japan; Department of Orthopaedic Surgery, Showa University Northern Yokohama Hospital, Yokohama, Japan; Department of Prosthodontics, Showa University School of Dentistry, Tokyo, Japan; Department of Oral Diagnostic Sciences, Division of Pathology, Showa University School of Dentistry, Tokyo, Japan

**Keywords:** Rheumatoid arthritis, Collagen antibody-induced arthritis, RANKL, Antibody, Inflammation, Articular cartilage

## Abstract

**Background:**

Rheumatoid arthritis (RA) is an inflammatory disease that leads to destruction of both articular cartilage and bone tissues. In rheumatic joints, synoviocytes and T-lymphocytes as well as bone cells produce the receptor activator of nuclear factor κ-B (RANK) ligand (RANKL), which binds to RANK on the surface of osteoclasts and their precursor cells to induce differentiation and activation of osteoclasts. Hence, inhibition of RANKL may be a promising approach to suppress osteolysis in RA. On the other hand, RANKL production by lymphocytes indicates the possibility that its inhibition would be effective to suppress inflammation in RA. In addition, it has been reported that cathepsin K, a predominant cysteine protease in osteoclasts, is involved in cartilage destruction in RA model mice. Here, we evaluated the effects of an anti-RANKL antibody on inflammation in footpads and degradation of articular cartilage in RA model mice.

**Results:**

We induced arthritis in mice by injection of anti-type II collagen antibodies and lipopolysaccharide (LPS). Inhibition of RANKL by an anti-RANKL antibody (OYC1, Oriental Yeast, Tokyo, Japan) was confirmed by increased bone volume in the metaphysis of tibias. Swelling in either limb until day 14 was seen in 5 of 6 mice injected with anti-collagen antibodies and LPS without treatment with OYC1, while that was seen in 4 of 5 mice treated with OYC1. The average arthritis scores on day 14 in those groups were 2.17 and 3.00, respectively, indicating that OYC1 did not ameliorate inflammation in the limbs. Histological analyses indicated that OYC1 does not protect articular cartilage from destruction in mice with arthritis.

**Conclusions:**

Our present study failed to show the effectiveness of an anti-RANKL antibody to ameliorate inflammation in the limbs or protect articular cartilage from degradation in a collagen antibody-induced arthritis mouse model.

**Electronic supplementary material:**

The online version of this article (doi:10.1186/s12952-014-0018-0) contains supplementary material, which is available to authorized users.

## Background

Rheumatoid arthritis (RA) is an inflammatory disease that leads to destruction of both articular cartilage and bone tissues. An articular cavity is a space enclosed by articular cartilage and synovial membranes. Single-layered cells compose a synovial membrane in normal joints, while in RA they proliferate in inflammatory conditions to form a pannus that destroys both cartilage and bone tissues [1]. It is well known that osteoblasts and/or osteocytes stimulated by physiological bone-resorbing factors such as activated vitamin D and parathyroid hormone produce the receptor activator of nuclear factor κ-B (RANK) ligand (RANKL), which binds to RANK distributed on the plasma membrane of osteoclasts and their precursor monocytes/macrophages. Interaction between RANKL and RANK induces differentiation and activation of osteoclasts [2,3]. Inflammatory cytokines including tumor necrosis factor-α, interleukin-1, interleukin-6, and interleukin-17 induce the expression of RANKL in osteoblasts, which augments bone destruction by osteoclasts in inflammatory conditions [4]. In addition, synovial fibroblasts and activated T lymphocytes abundantly produce RANKL, which is considered to contribute to osteoclastogenesis in RA [5]. Therefore, inhibition of the interaction between RANKL and RANK may be a promising approach to suppress osteolysis in RA. Denosumab, a fully human monoclonal antibody against RANKL, has been used clinically for treatment of osteoporosis and bone erosion associated with multiple myeloma and bone metastasis from tumors [6,7]. In addition, clinical studies on the effectiveness of denosumab against RA revealed that denosumab suppressed bone erosion [8].

The RANKL/RANK system functions not only in bones, but also in various tissues and cells including the immune system, vascular system, skin, and central nervous system [9]. The existence of this system in the immune system suggests that inhibition of RANKL can suppress the onset of RA and consequently inhibit cartilage degradation. It has been also reported that cathepsin K, a predominant cysteine protease in osteoclasts, is expressed in osteoclasts in contact with articular cartilage in RA patients and stabilized by glycosaminoglycans, such as chondroitin sulfate, which are abundantly produced by chondrocytes [10,11]. It was also reported that inhibition of cathepsin K suppressed cartilage degradation in collagen-induced arthritis in mice [12]. In addition, a clinaical study regarding the effects of denosumab on RA showed a transient decrease in the blood level of C-terminal telopeptide of type II collagen [8]. These observations suggest that anti-RANKL antibodies may have potential to inhibit degradation of articular cartilage associated with RA.

In the present study, we evaluated the effects of an anti-RANKL antibody on inflammation in footpads and degradation of articular cartilage in collagen antibody-induced arthritis model mice.

## Results

### Induction of arthritis and validation of anti-RANKL antibody in mice

**Figure 1:**
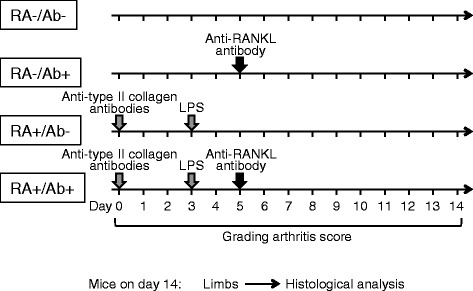
**Experimental design.** Eight-week-old male DBA1/J mice were divided into 4 groups (6 in each), i.e., RA-/Ab-, RA-/Ab+, RA+/Ab-, and RA+/Ab+. A cocktail of anti-type II collagen antibodies and *E. coli* LPS was injected intra-peritoneally into the RA+ mice on days 0 and 3, respectively. RA- mice were the control without injection of the anti-type II collagen antibodies and LPS. The OYC1 anti-RANKL monoclonal antibody (5 mg/kg) was injected subcutaneously into the Ab+ mice on day 5. Ab- mice were the control without injection the anti-RANKL antibody. Arthritis scores were determined daily from day 0 to 14. The 4 limbs were removed on day 14 for analysis of histological changes in the joints.

We examined the effects of an anti-RANKL antibody on inflammation in the joints and degradation of articular cartilage using a collagen antibody-induced arthritis mouse model. Male DBA1/J mice were divided into 4 experimental groups (6 in each), i.e., RA-/Ab-, RA-/Ab+, RA+/Ab-, and RA+/Ab+ (Figure 1). RA+ mice were injected with a cocktail of antibodies to type II collagen and lipopolysaccharide (LPS) (Chondrex, Inc., Redmond, WA) to induce arthritis. RA- mice were the control without injection of the collagen antibodies and LPS. Ab+ indicate that mice belonging to these groups were treated with the anti-RANKL antibody (OYC1, Oriental Yeast, Tokyo, Japan). Ab- mice are the control without treatment with the anti-RANKL antibody. Unfortunately, 1 of the 6 RA+/Ab+ mice died within 1 day after subcutaneous injection of the anti-RANKL antibody from peritonitis possibly caused by an inappropriate intra-peritoneal injection of the anesthetic agent.

**Figure 2:**
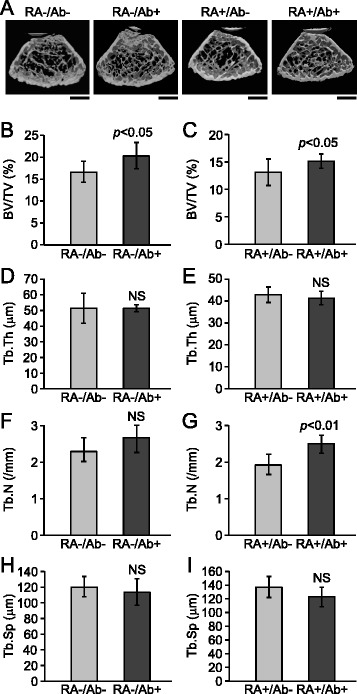
**Effect of anti-RANKL antibody OYC1 on morphology of tibias in RA- and RA+ mice.** Tibias excised from RA-/Ab-, RA-/Ab+, RA+/RA-, and RA+/Ab+ mice were subjected to three-dimensional micro-computed tomography. **(A)** Representative images of metaphysis of tibias. Bars: 600 μm. Trabecular bone volume (BV/TV) **(B**, **C)**, trabecular thickness (Tb.Th) **(D**, **E)**, trabecular number (Tb.N) **(F**, **G)**, and trabecular space (Tb.Sp) **(H, I)** in the metaphysis of tibias from RA- **(B**, **D**, **F**, **H)** and RA+ **(C**, **E**, **G**, **I)** mice was quantified. Data are shown as the mean ± SD (n = 6). Mann-Whitney *U*-test was used for statistical analysis. Significant differences are indicated in their *p*-values. NS shows that the difference between the groups is not significant.

μCT analyses indicated that administration of OYC1 anti-RANKL antibody increased bone mass both in tibias from RA- and RA+ mice (Figure 2A). The bone volume fraction (BV/TV) of trabecular bone in tibias from mice injected with the OYC1 anti-RANKL antibody (RA-/Ab+ mice) was significantly greater as compared to that in RA-/Ab- mice (Figure 2B). The same effect of the antibody was observed in RA+ mice (Figure 2C). Trabecular thickness (Tb.Th) was not affected by OYC-1 anti-RANKL antibody in either RA- or RA+ group (Figure 2D, E). While OYC1 antibody did not change trabecular number (Tb.N) in RA- mice (Figure 2F), the antibody significantly increased Tb.N in RA+ mice (Figure 2G). While trabecular space (Tb.Sp) tended to decline in mice received the anti-RANKL antibody in both RA- and RA+ groups, the effect of the antibody on Tb.Sp was not significant (Figure 2H, I). These quantitative μCT analyses indicated that the amount of OYC1 anti-RANKL antibody administered (5 mg/kg) was sufficient for inhibition of RANKL in our experimental model, as previously reported [13].

### Ineffectiveness of anti-RANKL antibody on inflammation induced by anti-collagen antibodies

We measured the footpad thickness of 4 limbs daily after injection of a cocktail of antibodies to type II collagen. Additional file 1: Figure S1 shows change in the thickness of each paw of the individual mice. Thickness of footpads was hardly changed in RA- mice in either Ab- or Ab+ group (Additional file 1: Figure S1A, C, E, G). On the other hand, some of the mice injected with a cocktail of antibodies to type II collagen on day 0 and LPS on day 3 showed swelling in the paws, especially those in fore limbs of both Ab- and Ab+ mice (Additional file 1: Figure S1B, D). Change in the thickness of hind paws was small (Additional file 1: Figure S1F, H). Then we summarized the frequency of swelling in each paw of the individual mouse observed 14 days after injection of anti-type II collagen antibodies (Table 1). Swelling frequency in either limb until day 14 in the RA+/Ab- group occurred in 5 of the 6 mice, whereas that was seen in 4 of 5 in the RA+/Ab+ group.

**Table 1:** **Frequency of swelling in limb pads**

**Group**	**Fore limb**		**Hind limb**		**Total**
	**Left**	**Right**	**Left**	**Right**	
RA-/Ab-	0/6	0/6	0/6	0/6	0/6
RA-/Ab+	0/6	0/6	0/6	0/6	0/6
RA+/Ab-	1/6	5/6	1/6	0/6	5/6
RA+/Ab+	4/5	3/5	1/5	1/5	4/5

Development of inflammation in the 4 limbs was evaluated daily by measuring the thickness of each footpad (Additional file 1: Figure S1). Occurrence of swelling in the limbs until day 14 is shown as a fraction, with the numerator and denominator representing the number of mice with swollen limb(s) and total number of mice in the group, respectively.

**Figure 3:**
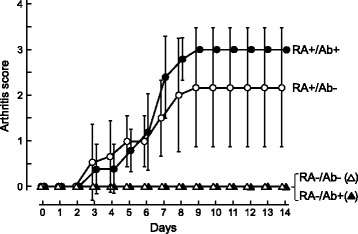
**Change in arthritis score after injection of antibodies to type II collagen.** Arthritis scores were determined daily after injection of a cocktail of antibodies to type II collagen according to the following criteria: 0, normal; 1, mild but definite redness and swelling of the ankle or wrist, or apparent redness and swelling limited to individual digits regardless of the number of affected digits; 2, moderate redness and swelling of ankle and wrist; 3, severe redness and swelling of the entire paw including digits; 4, maximally inflamed limb with involvement of multiple joints. Data are expressed as the mean ± SD. There was no statistical difference between RA+/Ab- group and RA+/Ab+ group (Steel-Dwass test).

We also determined the arthritis score of mice everyday as an index of severity of inflammation based on the frequency and extent of swelling and reddening in the limbs. The RA score for RA- mice did not increase irrespective of whether they were given anti-RANKL antibody (RA-/Ab+) or not (RA-/Ab-). On the other hand, the average arthritis score for the RA+/Ab- mice on day 12 was 2.17 ± 1.33 (mean ± SD), while that for the RA+/Ab+ mice was 3.00 ± 0 (Figure 3). These results show that administration of the anti-RANKL antibody did not have an effect to suppress inflammation induced by antibodies against type II collagen and LPS.

**Figure 4:**
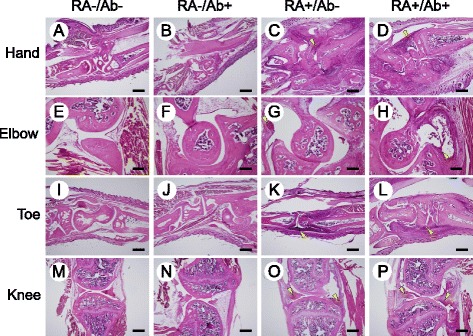
**Histological analyses of limb joints.** The 4 limbs were excised from all mice at 14 days after injection of a cocktail of antibodies to type II collagen. Decalcified 8-μm sections of hands **(A-D)**, elbows **(E-F)**, toes **(I-L)**, and knees **(M-P)** from RA-/Ab- **(A**, **E**, **I**, **M)**, RA-/Ab+ **(B**, **F**, **J**, **N)**, RA+/Ab- **(C**, **G**, **K**, **O)**, and RA+/Ab+ **(D**, **H**, **L**, **P)** mice were stained with hematoxylin and eosin. Synovial thickening and accumulation of inflammatory cells were observed in joints from the RA+ mice (arrowheads in panels **C**, **D**, **G**, **H**, **K**, **L**. **O**, and **P**). Bars: 500 μm **(A-D**, **I-P** **)** and 200 μm **(E-H)**.

Representative histological images of limb joints are shown in Figure 4. Synovial thickening, one of the typical changes seen in joints affected by RA, was observed in joints from both the RA+/Ab- (Figure 4C, G, K, O) and RA+/Ab+ (Figure 4D, H, L, P) mice. No significant difference was seen in regard to synovial thickening or infiltration of inflammatory cells in the RA+/Ab- mice and RA+/Ab+ mice, indicating that synovitis was not ameliorated by administration of the anti-RANKL antibody.

### Ineffectiveness of anti-RANKL antibody on degradation of cartilage induced by anti-collagen antibodies

**Figure 5:**
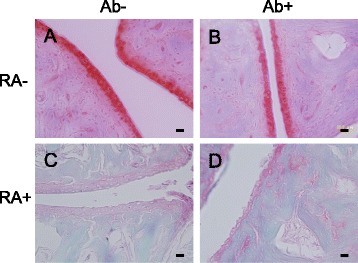
**Effect of anti-RANKL antibody OYC1 on cartilage erosion induced by collagen antibodies. (A-D)** Representative images of articular cartilage of joints in hands from RA-/Ab- **(A)**, RA-/Ab+ **(B)**, RA+/Ab- **(C)**, and RA+/AB+ **(D)** mice were stained with Fast Green and Safranin O. Note that cartilage matrix stained pink-red by Safranin O diminished and the erosion on the surface of cartilage was observed in the joints from RA+/Ab- and RA+/Ab+ mice. Bars: 20 μm.

Representative magnified images of articular cartilage from forelimbs of the RA-/Ab-, RA-/Ab+, RA+/Ab-, and RA+/Ab+ mice are shown in Figures 5A-D. The articular cartilage surface in the RA-/Ab- and RA-/Ab+ mice had a smooth and clean appearance (Figure 5A, B), whereas erosion was evident in that from RA+ mice irrespective of anti-RANKL antibody administration (Figure 5C, D). Cartilage matrix stained with Safranin O was reduced in RA+ mice (Figure 5C, D) in comparison with that in RA- mice (Figure 5A, B). Anti-RANKL antibody did not ameliorate the loss of cartilage matrix in RA+ mice (Figure 5C, D). These findings indicated that the OYC1 anti-RANKL antibody did not provide a protective effect on cartilage in the present collagen antibody-induced arthritis model.

## Discussion

Denosumab, a fully human monoclonal antibody against RANKL, is used clinically for treatment of osteoporosis and bone erosion associated with multiple myelomas and bone metastasis from those tumors [6,7]. In addition, the effectiveness of denosumab for treatment of bone erosion in RA is promising. On the other hand, degradation of articular cartilage is another serious problem encountered in patients with RA. Since it is known that RANKL participates in immunological reactions [14] and cathepsin K, one of the major proteases secreted by osteoclasts, is involved in cartilage degradation [12], it is important to determine if inhibition of RANKL can ameliorate cartilage degradation in RA. In the present study, we examined the effects of an anti-RANKL antibody on inflammation and cartilage degradation in a mouse model of RA induced by injection of a cocktail of antibodies to type II collagen.

We employed an RA model in mice produced by injection of antibodies against type II collagen and LPS to evaluate effectiveness of treatment with anti-RANKL antibody. Swelling of the paws (Table 1 and Additional file 1: Figure S1), increment in the RA score (Figure 3), and accumulation of inflammatory cells in the joints (Figure 4) indicated that inflammation was induced in the limbs by the treatments with antibodies against type II collagen and LPS. Reduced staining with Safranin O and the rough surface of the articular cartilage also suggested the degradation of the cartilage matrix in the joints in RA+ mice (Figure 5). Hence it is considered to be appropriate to use this model for evaluation of the effect of anti-RANKL antibody on inflammation and cartilage degeneration in RA.

It was previously reported that the OYC1 anti-RANKL antibody was effective to suppress osteoclast formation in mice from 1 to at least 28 days after subcutaneous injection at a dose of 5 mg/kg of body weight [13]. In the present study, the value for BV/TV in the metaphysis of tibias was significantly increased by subcutaneous injection of the same dose of the OYC1 anti-RANKL antibody (Figure 2B, C), demonstrating that the activity of RANKL was also suppressed by OYC1 in our experimental model. Hence, the inability of this antibody to reduce inflammation in synovial membranes and degradation of articular cartilage is not due to insufficient inhibition of RANKL. Osteoprotegerin (OPG), a soluble decoy receptor for RANKL, is secreted by osteoblasts and other types of cells and inhibits interaction between RANKL and RANK by binding to the former [15,16]. There are several reports of the effects of OPG administration on the pathogenesis of RA in animal models [17-20], including findings that bone erosion and osteoclast functions were down-regulated by treatment with OPG, whereas that did not have a significant effect on inflammation or cartilage erosion [20]. Those reports and the present results indicate that the contribution of RANKL to inflammation and cartilage erosion in RA is small.

Although human studies are required for evaluation of the effects of denosumab on cartilage erosion, the present animal study findings suggest that inhibition of RANKL by its antibodies is not effective for protection of articular cartilage from degradation associated with RA.

## Conclusion

The anti-RANKL antibody OYC1 did not protect articular cartilage from degradation in collagen antibody-induced arthritis model mice, despite inhibition of RANKL by the antibody shown by increased tibia bone volume.

## Materials and methods

### Reagents

An Arthrogen-CIA® 5-clone cocktail kit with LPS from *Escherichia coli* O111:B4 (Chondrex) was used for induction of collagen antibody-induced arthritis in mice. The anti-mouse RANKL monoclonal antibody (OYC1) was obtained from Oriental Yeast Co., Ltd. An animal COMP ELISA kit was purchased from AnaMar AB (Göteborg, Sweden). All other chemicals and reagents were obtained from commercial sources.

### Mice and experimental groups

Eight-week-old male DBA1/J mice (Japan SLC, Inc., Shizuoka, Japan) were randomly divided into 4 groups (6 in each), i.e., RA-/Ab-, RA-/Ab+, RA+/Ab-, and RA+/Ab+ (Table 1). Mice in the RA+ groups were given an intra-peritoneal injection of a cocktail of 5 clones of mouse monoclonal anti-type II collagen antibodies (1.5 mg/0.15 mL/head) on day 0, followed by an intra-peritoneal injection of *E. coli* LPS (50 μg/0.1 mL/head) on day 3 according to the manufacturer’s instruction (Chondrex). RA- mice were the control without injection of the anti-type II collagen antibodies and LPS. The OYC1 anti-RANKL monoclonal antibody (5 mg/kg body weight) was injected subcutaneously into mice in the Ab+ groups on day 5, while mice in the Ab- groups were not given that treatment. All mice were housed in a specific pathogen-free environment, and given free access to food and water. All experiments performed in this study were approved by the Ethical Board for Animal Experiments of Showa University, Tokyo, Japan (approval number: 14011).

### Scoring of RA

We observed mice daily for development of inflammation in the 4 limbs from day 0 to 14. The thickness of each footpad was measured using a slide caliper. In addition, the severity of inflammation was scored according to the criteria of Chondrex, as follows: 0, normal; 1, mild but definite redness and swelling of the ankle or wrist, or apparent redness and swelling limited to individual digits regardless of the number of affected digits; 2, moderate redness and swelling of ankle and wrist; 3, severe redness and swelling of the entire paw including digits; 4, maximally inflamed limb with involvement of multiple joints.

### X-ray micro-tomography

After blood sampling, all 4 limbs were dissected and fixed in 4% paraformaldehyde (pH 7.4). The tibias were subjected to three-dimensional micro-computed tomography. Three-dimensional microstructural image data obtained were reconstructed using TRI/3D-BON software (Ratoc System Engineering Co., Ltd., Tokyo, Japan).

### Histological examinations

The fixed limbs were decalcified in a decalcifying solution from Wako Pure Chemicals Co., Ltd. (Osaka, Japan) and embedded in paraffin. They were finally sliced into 8-μm sections, stained with hematoxylin and eosin, and observed under a microscope. For observation of the articular cartilage, the sections were stained with 0.1% Fast Green for 5 minutes, rinsed with 1% acetic acid for 10 seconds, and stained with Safranin O for 5 minutes.

### Statistical analyses

Results are expressed as the mean ± SD. A Mann-Whitney *U* test and Steel-Dwass test were used for statistical analyses, with *P* values less than 0.05 considered to be significant.

## Additional file

Additional file 1: Figure S1. Effect of anti-RANKL antibody on change in the thickness of footpads in mice with or without injection of a cocktail of anti-type II collagen antibodies. DBA1/J mice in the RA+ groups were given an intra-peritoneal injection of a cocktail of 5 clones of mouse monoclonal anti-type II collagen antibodies (Chondrex) on day 0, followed by an intra-peritoneal injection of *E. coli* LPS on day 3. RA- mice were the control without injection of the anti-type II collagen antibodies and LPS. The OYC1 anti-RANKL monoclonal antibody was injected subcutaneously into mice in the Ab+ groups on day 5, while mice in the Ab- groups were not given that treatment. The thickness of each footpad was measured daily from day 0 to day 14. Change in the thickness of each footpad was plotted as a line for individual mice. The left panels show the results from RA- mice (A, C, E, G), and the right panels are those from RA+ mice (B, D, F, H). Dotted and solid lines indicate the values obtained in Ab- and Ab+ mice, respectively. Results from left front paws (A, B), right front paws (C, D), left hind paws (E, F), and right hind paws (G, H) were indicated as separate figures.

## Abbreviations

RA: Rheumatoid arthritis
RANK: Receptor activator of nuclear factor κ-B
RANKL: RANK ligand
LPS: Lipopolysaccharide
BV/TV: Bone volume fraction
OPG: Osteoprotegerin
