# Supplementary material for: Effects of antibody to receptor activator of nuclear factor κ-B ligand on inflammation and cartilage degradation in collagen antibody-induced arthritis in mice
Source: J Negat Results Biomed. 2014 Dec 12;13:18. doi: 10.1186/s12952-014-0018-0 (PMC4272563; doi:10.1186/s12952-014-0018-0)
Supplement: Additional file 1: Figure S1. — Effect of anti-RANKL antibody on change in the thickness of footpads in mice with or without injection of a cocktail of anti-type II collagen antibodies. DBA1/J mice in the RA+ groups were given an intra-peritoneal injection of a cocktail of 5 clones of mouse monoclonal anti-type II collagen antibodies (Chondrex) on day 0, followed by an intra-peritoneal injection of E. coli LPS on day 3. RA- mice were the control without injection of the anti-type II collagen antibodies and LPS. The OYC1 anti-RANKL monoclonal antibody was injected subcutaneously into mice in the Ab+ groups on day 5, while mice in the Ab- groups were not given that treatment. The thickness of each footpad was measured daily from day 0 to day 14. Change in the thickness of each footpad was plotted as a line for individual mice. The left panels show the results from RA- mice (A, C, E, G), and the right panels are those from RA+ mice (B, D, F, H). Dotted and solid lines indicate the values obtained in Ab- and Ab+ mice, respectively. Results from left front paws (A, B), right front paws (C, D), left hind paws (E, F), and right hind paws (G, H) were indicated as separate figures. [file 12952_2014_18_MOESM1_ESM.pptx]

## Slide 1
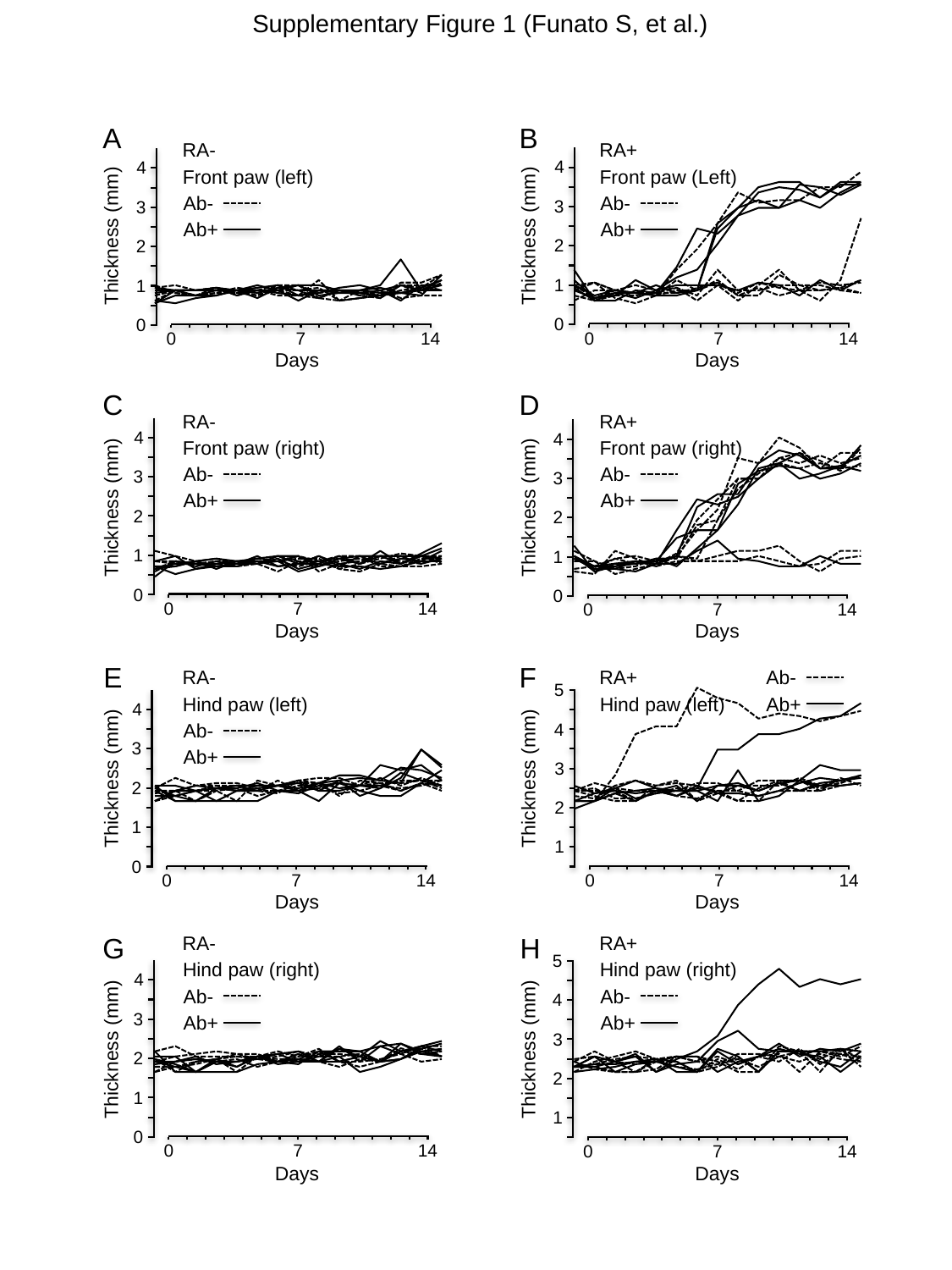

Supplementary Figure 1 (Funato S, et al.)
A
B
### Chart
| Category | RA(-）、抗体（-） マウス１ | RA(-）、抗体（-） マウス２ | RA(-）、抗体（-） マウス３ | RA(-）、抗体（-） マウス４ | RA(-）、抗体（-） マウス５ | RA(-）、抗体（-） マウス６ | RA(-）、抗体（+） マウス１ | RA(-）、抗体（+） マウス２ | RA(-）、抗体（+） マウス３ | RA(-）、抗体（+） マウス４ | RA(-）、抗体（+） マウス５ | RA(-）、抗体（+） マウス６ |
|---|---|---|---|---|---|---|---|---|---|---|---|---|
| 0day | 1.45 | 1.2 | 1.5 | 1.3 | 1.3 | 1.45 | 1.35 | 1.45 | 1.4 | 1.15 | 1.15 | 1.2 |
| 1day | 1.5 | 1.4 | 1.35 | 1.4 | 1.4 | 1.35 | 1.4 | 1.4 | 1.4 | 1.3 | 1.4 | 1.15 |
| 2days | 1.4 | 1.4 | 1.3 | 1.3 | 1.4 | 1.3 | 1.4 | 1.3 | 1.4 | 1.3 | 1.4 | 1.25 |
| 3days | 1.45 | 1.4 | 1.45 | 1.35 | 1.45 | 1.35 | 1.4 | 1.3 | 1.45 | 1.4 | 1.4 | 1.3 |
| 4days | 1.3 | 1.35 | 1.4 | 1.35 | 1.4 | 1.45 | 1.4 | 1.4 | 1.3 | 1.4 | 1.4 | 1.4 |
| 5days | 1.4 | 1.4 | 1.35 | 1.3 | 1.3 | 1.4 | 1.4 | 1.5 | 1.4 | 1.35 | 1.45 | 1.25 |
| 6days | 1.3 | 1.5 | 1.4 | 1.5 | 1.4 | 1.4 | 1.4 | 1.4 | 1.35 | 1.45 | 1.5 | 1.45 |
| 7days | 1.3 | 1.5 | 1.5 | 1.4 | 1.4 | 1.3 | 1.2 | 1.5 | 1.3 | 1.4 | 1.3 | 1.5 |
| 8days | 1.25 | 1.35 | 1.25 | 1.25 | 1.45 | 1.6 | 1.4 | 1.3 | 1.35 | 1.4 | 1.4 | 1.5 |
| 9days | 1.2 | 1.4 | 1.4 | 1.4 | 1.2 | 1.2 | 1.4 | 1.35 | 1.45 | 1.35 | 1.4 | 1.4 |
| 10days | 1.25 | 1.35 | 1.3 | 1.3 | 1.4 | 1.25 | 1.4 | 1.4 | 1.5 | 1.35 | 1.4 | 1.35 |
| 11days | 1.3 | 1.25 | 1.25 | 1.4 | 1.3 | 1.3 | 1.45 | 1.35 | 1.4 | 1.3 | 1.5 | 1.4 |
| 12days | 1.55 | 1.5 | 1.5 | 1.25 | 1.4 | 1.5 | 1.35 | 1.35 | 1.2 | 1.35 | 2.0 | 1.5 |
| 13days | 1.55 | 1.35 | 1.5 | 1.3 | 1.45 | 1.45 | 1.3 | 1.4 | 1.5 | 1.45 | 1.4 | 1.4 |
| 14days | 1.7 | 1.55 | 1.4 | 1.3 | 1.7 | 1.4 | 1.7 | 1.6 | 1.5 | 1.6 | 1.5 | 1.4 |4
3
2
1
0
0
7
14
Thickness (mm)
Days
### Chart
| Category | RA(+）、抗体（-） マウス１ | RA(+）、抗体（-） マウス２ | RA(+）、抗体（-） マウス３ | RA(+）、抗体（-） マウス４ | RA(+）、抗体（-） マウス５ | RA(+）、抗体（-） マウス６ | RA(+)、抗体(+) マウス２ | RA(+)、抗体(+) マウス３ | RA(+)、抗体(+) マウス４ | RA(+)、抗体(+) マウス５ | RA(+)、抗体(+) マウス６ |
|---|---|---|---|---|---|---|---|---|---|---|---|
| 0day | 1.4 | 1.3 | 1.45 | 1.5 | 1.2 | 1.5 | 1.4 | 1.8 | 1.45 | 1.6 | 1.5 |
| 1day | 1.55 | 1.2 | 1.55 | 1.55 | 1.4 | 1.25 | 1.25 | 1.2 | 1.2 | 1.25 | 1.3 |
| 2days | 1.4 | 1.3 | 1.25 | 1.4 | 1.4 | 1.4 | 1.35 | 1.2 | 1.35 | 1.3 | 1.4 |
| 3days | 1.35 | 1.3 | 1.15 | 1.35 | 1.5 | 1.35 | 1.35 | 1.4 | 1.25 | 1.6 | 1.35 |
| 4days | 1.3 | 1.35 | 1.3 | 1.3 | 1.4 | 1.35 | 1.3 | 1.35 | 1.4 | 1.4 | 1.5 |
| 5days | 1.6 | 1.35 | 1.8 | 1.4 | 1.45 | 1.45 | 1.3 | 1.85 | 1.65 | 1.5 | 1.35 |
| 6days | 1.4 | 1.445 | 2.2 | 1.4 | 1.3 | 1.2 | 1.4 | 2.6 | 1.8 | 1.5 | 1.4 |
| 7days | 1.55 | 1.55 | 2.7 | 1.6 | 1.8 | 1.5 | 2.6 | 2.5 | 2.3 | 1.5 | 2.7 |
| 8days | 1.3 | 1.35 | 3.3 | 1.3 | 1.4 | 1.2 | 3.0 | 2.85 | 2.85 | 1.4 | 3.0 |
| 9days | 1.45 | 1.55 | 3.1 | 1.3 | 1.4 | 1.5 | 3.15 | 3.3 | 3.0 | 1.55 | 3.4 |
| 10days | 1.3 | 1.45 | 3.15 | 1.7 | 1.5 | 1.8 | 3.0 | 3.4 | 3.0 | 1.5 | 3.5 |
| 11days | 1.4 | 1.4 | 3.15 | 1.5 | 1.5 | 1.4 | 3.45 | 3.35 | 3.15 | 1.3 | 3.5 |
| 12days | 1.4 | 1.55 | 3.4 | 1.5 | 1.4 | 1.2 | 3.4 | 3.2 | 3.0 | 1.6 | 3.2 |
| 13days | 1.45 | 1.5 | 3.4 | 1.4 | 1.45 | 1.6 | 3.25 | 3.5 | 3.3 | 1.4 | 3.45 |
| 14days | 1.6 | 1.55 | 3.7 | 1.35 | 1.35 | 2.8 | 3.45 | 3.5 | 3.5 | 1.6 | 3.45 |4
3
2
1
0
0
7
14
Thickness (mm)
Days
RA-
Front paw (left)
Ab-
Ab+
RA+
Front paw (Left)
Ab-
Ab+
C
D
### Chart
| Category | RA(-）、抗体（-） マウス１ | RA(-）、抗体（-） マウス２ | RA(-）、抗体（-） マウス３ | RA(-）、抗体（-） マウス４ | RA(-）、抗体（-） マウス５ | RA(-）、抗体（-） マウス６ | RA(-）、抗体（+） マウス１ | RA(-）、抗体（+） マウス２ | RA(-）、抗体（+） マウス３ | RA(-）、抗体（+） マウス４ | RA(-）、抗体（+） マウス５ | RA(-）、抗体（+） マウス６ |
|---|---|---|---|---|---|---|---|---|---|---|---|---|
| 0day | 1.6 | 1.4 | 1.4 | 1.2 | 1.25 | 1.4 | 1.25 | 1.4 | 1.3 | 1.25 | 1.1 | 1.3 |
| 1day | 1.5 | 1.4 | 1.35 | 1.4 | 1.4 | 1.4 | 1.35 | 1.5 | 1.3 | 1.3 | 1.4 | 1.15 |
| 2days | 1.4 | 1.35 | 1.35 | 1.35 | 1.3 | 1.3 | 1.4 | 1.25 | 1.4 | 1.4 | 1.35 | 1.25 |
| 3days | 1.45 | 1.35 | 1.35 | 1.4 | 1.4 | 1.4 | 1.45 | 1.35 | 1.3 | 1.25 | 1.3 | 1.3 |
| 4days | 1.35 | 1.35 | 1.35 | 1.4 | 1.35 | 1.3 | 1.4 | 1.4 | 1.3 | 1.4 | 1.35 | 1.35 |
| 5days | 1.35 | 1.4 | 1.45 | 1.4 | 1.45 | 1.35 | 1.4 | 1.45 | 1.4 | 1.35 | 1.5 | 1.35 |
| 6days | 1.4 | 1.4 | 1.5 | 1.4 | 1.4 | 1.2 | 1.3 | 1.5 | 1.4 | 1.5 | 1.3 | 1.45 |
| 7days | 1.3 | 1.5 | 1.4 | 1.5 | 1.45 | 1.4 | 1.35 | 1.5 | 1.2 | 1.35 | 1.35 | 1.25 |
| 8days | 1.4 | 1.4 | 1.3 | 1.2 | 1.45 | 1.4 | 1.5 | 1.3 | 1.3 | 1.35 | 1.5 | 1.35 |
| 9days | 1.45 | 1.5 | 1.4 | 1.35 | 1.25 | 1.25 | 1.35 | 1.45 | 1.45 | 1.3 | 1.3 | 1.5 |
| 10days | 1.35 | 1.5 | 1.45 | 1.4 | 1.35 | 1.2 | 1.25 | 1.5 | 1.35 | 1.3 | 1.5 | 1.35 |
| 11days | 1.45 | 1.3 | 1.35 | 1.5 | 1.5 | 1.35 | 1.4 | 1.5 | 1.5 | 1.25 | 1.35 | 1.6 |
| 12days | 1.55 | 1.3 | 1.3 | 1.4 | 1.5 | 1.5 | 1.35 | 1.5 | 1.35 | 1.3 | 1.45 | 1.35 |
| 13days | 1.5 | 1.45 | 1.3 | 1.35 | 1.5 | 1.5 | 1.55 | 1.35 | 1.5 | 1.4 | 1.4 | 1.5 |
| 14days | 1.4 | 1.45 | 1.35 | 1.5 | 1.35 | 1.5 | 1.75 | 1.45 | 1.65 | 1.4 | 1.6 | 1.45 |4
3
2
1
0
0
7
14
Thickness (mm)
Days
### Chart
| Category | RA(+）、抗体（-） マウス１ | RA(+）、抗体（-） マウス２ | RA(+）、抗体（-） マウス３ | RA(+）、抗体（-） マウス４ | RA(+）、抗体（-） マウス５ | RA(+）、抗体（-） マウス６ | RA(+)、抗体(+) マウス２ | RA(+)、抗体(+) マウス３ | RA(+)、抗体(+) マウス４ | RA(+)、抗体(+) マウス５ | RA(+)、抗体(+) マウス６ |
|---|---|---|---|---|---|---|---|---|---|---|---|
| 0day | 1.2 | 1.25 | 1.4 | 1.45 | 1.6 | 1.5 | 1.45 | 1.5 | 1.5 | 1.7 | 1.45 |
| 1day | 1.15 | 1.3 | 1.4 | 1.3 | 1.4 | 1.25 | 1.3 | 1.3 | 1.2 | 1.2 | 1.25 |
| 2days | 1.6 | 1.3 | 1.15 | 1.45 | 1.25 | 1.45 | 1.25 | 1.353 | 1.3 | 1.35 | 1.25 |
| 3days | 1.45 | 1.4 | 1.25 | 1.35 | 1.3 | 1.5 | 1.2 | 1.4 | 1.35 | 1.35 | 1.4 |
| 4days | 1.3 | 1.3 | 1.35 | 1.4 | 1.45 | 1.4 | 1.35 | 1.35 | 1.4 | 1.45 | 1.35 |
| 5days | 1.5 | 1.4 | 1.55 | 1.5 | 1.45 | 1.5 | 1.5 | 2.0 | 1.85 | 1.3 | 1.35 |
| 6days | 1.4 | 1.4 | 2.2 | 1.45 | 2.1 | 2.0 | 2.45 | 2.6 | 2.0 | 1.65 | 1.6 |
| 7days | 1.4 | 1.5 | 2.6 | 2.2 | 2.2 | 2.4 | 2.7 | 2.5 | 2.0 | 2.0 | 1.8 |
| 8days | 1.4 | 1.6 | 3.0 | 2.8 | 3.0 | 3.4 | 2.7 | 2.65 | 2.9 | 2.5 | 1.45 |
| 9days | 1.5 | 1.6 | 3.0 | 3.1 | 3.0 | 3.3 | 3.3 | 3.0 | 3.15 | 3.2 | 1.4 |
| 10days | 1.4 | 1.7 | 3.4 | 3.4 | 3.3 | 3.8 | 3.55 | 3.3 | 3.25 | 3.3 | 1.3 |
| 11days | 1.3 | 1.4 | 3.3 | 3.5 | 3.2 | 3.6 | 3.45 | 3.0 | 3.2 | 3.5 | 1.3 |
| 12days | 1.35 | 1.2 | 3.45 | 3.35 | 3.3 | 3.2 | 3.2 | 3.1 | 3.0 | 3.2 | 1.5 |
| 13days | 1.6 | 1.45 | 3.3 | 3.15 | 3.2 | 3.5 | 3.2 | 3.25 | 3.1 | 3.25 | 1.35 |
| 14days | 1.6 | 1.5 | 3.4 | 3.6 | 3.25 | 3.5 | 3.65 | 3.45 | 3.3 | 3.15 | 1.35 |4
3
2
1
0
0
7
14
Thickness (mm)
Days
RA-
Front paw (right)
Ab-
Ab+
RA+
Front paw (right)
Ab-
Ab+
E
F
RA-
Hind paw (left)
Ab-
Ab+
RA+
Ab-
Hind paw (left)
Ab+
### Chart
| Category | RA(+）、抗体（-） マウス１ | RA(+）、抗体（-） マウス２ | RA(+）、抗体（-） マウス３ | RA(+）、抗体（-） マウス４ | RA(+）、抗体（-） マウス５ | RA(+）、抗体（-） マウス６ | RA(+)、抗体(+) マウス２ | RA(+)、抗体(+) マウス３ | RA(+)、抗体(+) マウス４ | RA(+)、抗体(+) マウス５ | RA(+)、抗体(+) マウス６ |
|---|---|---|---|---|---|---|---|---|---|---|---|
| 0day | 2.2 | 2.2 | 2.2 | 2.0 | 2.2 | 2.1 | 2.0 | 2.0 | 1.85 | 2.3 | 2.0 |
| 1day | 2.2 | 2.25 | 2.1 | 2.0 | 2.35 | 2.05 | 2.0 | 2.0 | 2.0 | 2.15 | 2.15 |
| 2days | 2.2 | 2.05 | 2.0 | 2.5 | 2.25 | 2.3 | 2.15 | 2.15 | 2.25 | 2.25 | 2.2 |
| 3days | 2.0 | 2.05 | 2.0 | 3.3 | 2.2 | 2.4 | 2.2 | 2.0 | 2.4 | 2.05 | 2.15 |
| 4days | 2.2 | 2.15 | 2.3 | 3.45 | 2.2 | 2.3 | 2.25 | 2.2 | 2.25 | 2.15 | 2.2 |
| 5days | 2.1 | 2.25 | 2.35 | 3.45 | 2.2 | 2.4 | 2.2 | 2.3 | 2.1 | 2.2 | 2.1 |
| 6days | 2.05 | 2.25 | 2.3 | 4.2 | 2.35 | 2.0 | 2.2 | 2.0 | 2.25 | 2.2 | 2.3 |
| 7days | 2.2 | 2.2 | 2.15 | 4.0 | 2.35 | 2.15 | 2.3 | 2.3 | 3.0 | 2.0 | 2.15 |
| 8days | 2.0 | 2.2 | 2.0 | 3.9 | 2.2 | 2.3 | 2.3 | 2.35 | 3.0 | 2.6 | 2.15 |
| 9days | 2.25 | 2.1 | 2.0 | 3.6 | 2.4 | 2.3 | 2.2 | 2.2 | 3.3 | 2.0 | 2.1 |
| 10days | 2.4 | 2.2 | 2.4 | 3.7 | 2.4 | 2.3 | 2.35 | 2.35 | 3.3 | 2.1 | 2.2 |
| 11days | 2.4 | 2.2 | 2.4 | 3.65 | 2.2 | 2.45 | 2.4 | 2.2 | 3.4 | 2.4 | 2.35 |
| 12days | 2.2 | 2.2 | 2.25 | 3.55 | 2.3 | 2.3 | 2.3 | 2.35 | 3.6 | 2.7 | 2.45 |
| 13days | 2.45 | 2.3 | 2.35 | 3.65 | 2.4 | 2.35 | 2.3 | 2.4 | 3.65 | 2.6 | 2.4 |
| 14days | 2.3 | 2.35 | 2.5 | 3.75 | 2.5 | 2.5 | 2.35 | 2.45 | 3.9 | 2.6 | 2.5 |5
4
3
2
1
0
7
14
Thickness (mm)
Days
### Chart
| Category | RA(-）、抗体（-） マウス１ | RA(-）、抗体（-） マウス２ | RA(-）、抗体（-） マウス３ | RA(-）、抗体（-） マウス４ | RA(-）、抗体（-） マウス５ | RA(-）、抗体（-） マウス６ | RA(-）、抗体（+） マウス１ | RA(-）、抗体（+） マウス２ | RA(-）、抗体（+） マウス３ | RA(-）、抗体（+） マウス４ | RA(-）、抗体（+） マウス５ | RA(-）、抗体（+） マウス６ |
|---|---|---|---|---|---|---|---|---|---|---|---|---|
| 0day | 2.25 | 2.15 | 2.2 | 2.0 | 2.0 | 2.0 | 2.2 | 2.1 | 2.0 | 2.3 | 2.25 | 2.3 |
| 1day | 2.45 | 2.2 | 2.2 | 2.1 | 2.2 | 2.2 | 2.0 | 2.1 | 2.2 | 2.0 | 2.1 | 2.3 |
| 2days | 2.3 | 2.0 | 2.2 | 2.2 | 2.2 | 2.3 | 2.0 | 2.2 | 2.3 | 2.0 | 2.0 | 2.2 |
| 3days | 2.35 | 2.2 | 2.2 | 2.25 | 2.3 | 2.3 | 2.25 | 2.25 | 2.25 | 2.0 | 2.25 | 2.0 |
| 4days | 2.35 | 2.25 | 2.0 | 2.2 | 2.2 | 2.3 | 2.25 | 2.3 | 2.2 | 2.0 | 2.3 | 2.2 |
| 5days | 2.25 | 2.1 | 2.4 | 2.2 | 2.2 | 2.2 | 2.35 | 2.3 | 2.25 | 2.0 | 2.2 | 2.3 |
| 6days | 2.3 | 2.2 | 2.3 | 2.15 | 2.2 | 2.4 | 2.2 | 2.3 | 2.3 | 2.2 | 2.3 | 2.3 |
| 7days | 2.4 | 2.2 | 2.35 | 2.4 | 2.3 | 2.2 | 2.15 | 2.25 | 2.35 | 2.2 | 2.2 | 2.4 |
| 8days | 2.45 | 2.25 | 2.35 | 2.35 | 2.3 | 2.35 | 2.3 | 2.35 | 2.2 | 2.0 | 2.35 | 2.2 |
| 9days | 2.45 | 2.35 | 2.25 | 2.1 | 2.15 | 2.25 | 2.35 | 2.4 | 2.4 | 2.35 | 2.5 | 2.2 |
| 10days | 2.3 | 2.3 | 2.3 | 2.4 | 2.2 | 2.3 | 2.3 | 2.45 | 2.1 | 2.2 | 2.5 | 2.3 |
| 11days | 2.45 | 2.3 | 2.35 | 2.35 | 2.3 | 2.3 | 2.25 | 2.4 | 2.25 | 2.1 | 2.4 | 2.7 |
| 12days | 2.3 | 2.2 | 2.2 | 2.35 | 2.4 | 2.25 | 2.45 | 2.35 | 2.55 | 2.1 | 2.65 | 2.6 |
| 13days | 2.45 | 2.35 | 2.35 | 2.4 | 2.4 | 2.3 | 3.0 | 3.0 | 2.4 | 2.35 | 2.6 | 2.7 |
| 14days | 2.3 | 2.3 | 2.2 | 2.25 | 2.4 | 2.4 | 2.7 | 2.65 | 2.3 | 2.6 | 2.45 | 2.4 |4
3
2
1
0
0
7
14
Thickness (mm)
Days
G
H
RA-
Hind paw (right)
Ab-
Ab+
RA+
Hind paw (right)
Ab-
Ab+
### Chart
| Category | RA(-）、抗体（-） マウス１ | RA(-）、抗体（-） マウス２ | RA(-）、抗体（-） マウス３ | RA(-）、抗体（-） マウス４ | RA(-）、抗体（-） マウス５ | RA(-）、抗体（-） マウス６ | RA(-）、抗体（+） マウス１ | RA(-）、抗体（+） マウス２ | RA(-）、抗体（+） マウス３ | RA(-）、抗体（+） マウス４ | RA(-）、抗体（+） マウス５ | RA(-）、抗体（+） マウス６ |
|---|---|---|---|---|---|---|---|---|---|---|---|---|
| 0day | 2.4 | 2.0 | 2.25 | 2.1 | 2.0 | 2.2 | 2.3 | 2.25 | 2.15 | 2.4 | 2.2 | 2.2 |
| 1day | 2.5 | 2.2 | 2.0 | 2.1 | 2.1 | 2.3 | 2.3 | 2.15 | 2.2 | 2.0 | 2.1 | 2.2 |
| 2days | 2.3 | 2.25 | 2.2 | 2.15 | 2.2 | 2.35 | 2.0 | 2.0 | 2.25 | 2.0 | 2.0 | 2.3 |
| 3days | 2.3 | 2.2 | 2.25 | 2.25 | 2.25 | 2.4 | 2.2 | 2.2 | 2.2 | 2.0 | 2.25 | 2.15 |
| 4days | 2.3 | 2.25 | 2.0 | 2.35 | 2.3 | 2.35 | 2.1 | 2.2 | 2.2 | 2.0 | 2.1 | 2.2 |
| 5days | 2.3 | 2.1 | 2.3 | 2.35 | 2.25 | 2.25 | 2.3 | 2.3 | 2.3 | 2.15 | 2.3 | 2.25 |
| 6days | 2.4 | 2.2 | 2.25 | 2.2 | 2.2 | 2.3 | 2.15 | 2.2 | 2.2 | 2.2 | 2.35 | 2.35 |
| 7days | 2.3 | 2.2 | 2.25 | 2.3 | 2.25 | 2.4 | 2.2 | 2.15 | 2.35 | 2.25 | 2.2 | 2.4 |
| 8days | 2.45 | 2.35 | 2.2 | 2.4 | 2.4 | 2.3 | 2.2 | 2.4 | 2.2 | 2.2 | 2.3 | 2.2 |
| 9days | 2.2 | 2.35 | 2.1 | 2.2 | 2.2 | 2.3 | 2.45 | 2.4 | 2.3 | 2.2 | 2.45 | 2.5 |
| 10days | 2.1 | 2.3 | 2.25 | 2.2 | 2.3 | 2.35 | 2.3 | 2.4 | 2.0 | 2.3 | 2.4 | 2.2 |
| 11days | 2.2 | 2.2 | 2.2 | 2.25 | 2.2 | 2.2 | 2.6 | 2.5 | 2.1 | 2.2 | 2.2 | 2.5 |
| 12days | 2.46 | 2.4 | 2.4 | 2.35 | 2.4 | 2.25 | 2.4 | 2.35 | 2.25 | 2.25 | 2.55 | 2.55 |
| 13days | 2.35 | 2.35 | 2.5 | 2.2 | 2.45 | 2.5 | 2.5 | 2.5 | 2.4 | 2.45 | 2.35 | 2.4 |
| 14days | 2.4 | 2.4 | 2.3 | 2.25 | 2.3 | 2.5 | 2.4 | 2.6 | 2.3 | 2.55 | 2.3 | 2.45 |4
3
2
1
0
0
7
14
Thickness (mm)
Days
### Chart
| Category | RA(+）、抗体（-） マウス１ | RA(+）、抗体（-） マウス２ | RA(+）、抗体（-） マウス３ | RA(+）、抗体（-） マウス４ | RA(+）、抗体（-） マウス５ | RA(+）、抗体（-） マウス６ | RA(+)、抗体(+) マウス２ | RA(+)、抗体(+) マウス３ | RA(+)、抗体(+) マウス４ | RA(+)、抗体(+) マウス５ | RA(+)、抗体(+) マウス６ |
|---|---|---|---|---|---|---|---|---|---|---|---|
| 0day | 2.2 | 2.1 | 2.2 | 2.25 | 2.0 | 2.1 | 2.1 | 2.0 | 2.1 | 2.1 | 2.1 |
| 1day | 2.4 | 2.1 | 2.05 | 2.3 | 2.2 | 2.15 | 2.3 | 2.05 | 2.1 | 2.3 | 2.15 |
| 2days | 2.2 | 2.0 | 2.0 | 2.2 | 2.3 | 2.25 | 2.0 | 2.1 | 2.2 | 2.2 | 2.15 |
| 3days | 2.15 | 2.0 | 2.0 | 2.0 | 2.4 | 2.3 | 2.15 | 2.2 | 2.3 | 2.35 | 2.2 |
| 4days | 2.2 | 2.05 | 2.25 | 2.2 | 2.25 | 2.25 | 2.2 | 2.25 | 2.0 | 2.0 | 2.2 |
| 5days | 2.1 | 2.3 | 2.3 | 2.2 | 2.3 | 2.2 | 2.3 | 2.0 | 2.15 | 2.25 | 2.1 |
| 6days | 2.05 | 2.2 | 2.3 | 2.0 | 2.2 | 2.0 | 2.3 | 2.0 | 2.2 | 2.4 | 2.0 |
| 7days | 2.15 | 2.15 | 2.2 | 2.1 | 2.3 | 2.25 | 2.0 | 2.45 | 2.6 | 2.7 | 2.4 |
| 8days | 2.25 | 2.35 | 2.0 | 2.3 | 2.15 | 2.05 | 2.2 | 2.3 | 2.8 | 3.3 | 2.15 |
| 9days | 2.1 | 2.35 | 2.0 | 2.0 | 2.3 | 2.3 | 2.3 | 2.0 | 2.45 | 3.7 | 2.3 |
| 10days | 2.3 | 2.3 | 2.4 | 2.5 | 2.35 | 2.2 | 2.45 | 2.4 | 2.4 | 4.0 | 2.55 |
| 11days | 2.4 | 2.2 | 2.4 | 2.4 | 2.0 | 2.45 | 2.35 | 2.4 | 2.4 | 3.65 | 2.3 |
| 12days | 2.0 | 2.35 | 2.4 | 2.25 | 2.4 | 2.15 | 2.3 | 2.4 | 2.2 | 3.8 | 2.45 |
| 13days | 2.45 | 2.25 | 2.35 | 2.45 | 2.3 | 2.35 | 2.0 | 2.45 | 2.1 | 3.7 | 2.4 |
| 14days | 2.1 | 2.2 | 2.3 | 2.25 | 2.5 | 2.2 | 2.3 | 2.4 | 2.4 | 3.8 | 2.55 |5
4
3
2
1
0
7
14
Thickness (mm)
Days
